# The Increase in Paraoxonase 1 Is Associated With Decrease in Left Ventricular Volume in Kidney Transplant Recipients

**DOI:** 10.3389/fcvm.2021.763389

**Published:** 2021-12-02

**Authors:** Philip W. Connelly, Andrew T. Yan, Michelle M. Nash, Rachel M. Wald, Charmaine Lok, Lakshman Gunaratnam, Anish Kirpalani, G. V. Ramesh Prasad

**Affiliations:** ^1^Division of Endocrinology and Metabolism, Department of Medicine, St. Michael's Hospital, University of Toronto, Toronto, ON, Canada; ^2^Department of Laboratory Medicine and Pathobiology, University of Toronto, Toronto, ON, Canada; ^3^Keenan Research Centre for Biomedical Science of St. Michael's Hospital, Toronto, ON, Canada; ^4^Division of Cardiology, St. Michael's Hospital, University of Toronto, Toronto, ON, Canada; ^5^Kidney Transplant Program, St. Michael's Hospital, Toronto, ON, Canada; ^6^Division of Cardiology, Toronto General Hospital, University of Toronto, Toronto, ON, Canada; ^7^Division of Nephrology, Toronto General Hospital, University of Toronto, Toronto, ON, Canada; ^8^Division of Nephrology, London Health Sciences Centre, Western University, London, ON, Canada; ^9^Department of Medical Imaging, St. Michael's Hospital, University of Toronto, Toronto, ON, Canada

**Keywords:** paraoxonase 1, kidney transplantation, left ventricular hypertrophy, cardiac magnetic resonance imaging, dialysis

## Abstract

**Background:** Patients on dialysis have impaired cardiac function, in part due to increased fluid volume and ventricular stress. Restored kidney function through transplantation reduces left ventricular volume in both systole and diastole. We previously reported that the decrease in NT-proB-type natriuretic peptide (NT-proBNP) was associated with a decrease in adiponectin. Paraoxonase 1 (PON1) has been inversely associated with cardiovascular outcomes. We now report the association of changes in PON1 with changes in left ventricular volume and left ventricular mass after kidney transplantation.

**Design:** Patients on dialysis were assessed at baseline and 12 months after kidney transplantation (*n* = 38). A comparison group of patients on dialysis who were not expected to receive a transplant in the next 24 months were studied (*n* = 43) to determine if the change of PON1 with kidney transplantation achieved a significance greater than that due to biologic variation. Left ventricular volume and mass were determined by cardiac magnetic resonance imaging. PON1 was measured by arylesterase activity and by mass.

**Results:** PON1 mass and activity were not different between the groups at baseline. Both PON1 mass and activity were increased post-kidney transplantation (*p* < 0.0001 for change). The change in PON1 mass (*p* = 0.0062) and PON1 arylesterase activity (*p* = 0.0254) were inversely correlated with the change in NT-proBNP for patients receiving a kidney transplant. However, only the change in the PON1 mass, and not the change in PON1 arylesterase, was inversely correlated with the change in left ventricular volume (ml/m2.7) (*p* = 0.0146 and 0.0114 for diastolic and systolic, respectively) and with the change in hemoglobin (*p* = 0.0042).

**Conclusion:** Both PON1 mass and arylesterase activity are increased by kidney transplantation. The increase in PON1 mass is consistent with a novel relationship to the increase in hemoglobin and decrease in left ventricular volume and NT-proBNP seen when kidney function is restored.

## Introduction

Patients with chronic kidney disease (CKD) are at increased risk for cardiovascular disease, including non-atherosclerotic disease characterized by left ventricular hypertrophy (LVH) (1). Among the noninvasive tools used to measure left ventricular (LV) volume and mass, cardiac magnetic resonance imaging (CMR) is particularly useful due to its accuracy and precision. We recently reported LV changes by CMR in patients with end-stage kidney disease (ESKD) in whom kidney function was partially restored in the form of a kidney transplant (KT) (2). In addition, LV changes by CMR can be correlated with cardiovascular biomarkers such as adiponectin (2). While both LV volume and adiponectin decreased with KT, change in adiponectin was not associated with change in LV volume (2). Since CMR is a useful tool to correlate cardiovascular biomarker changes with both changes in kidney function after KT and measurable changes in cardiac function, we extended our study to paraoxonase 1 (PON1).

PON1 is a 43kDa protein synthesized primarily by hepatocytes and released to high density lipoproteins (HDL). PON1 has both metal binding and lactonase properties (3). Homocysteine thiolactone has been identified as a substrate for PON1 (4). Methylation of the PON1 gene has been associated with clopidogrel resistance (5).

HDL enriched in PON1 has been associated with less vascular disease in type 1 diabetes (6). The first report that PON1 activity was reduced in patients with ESKD on hemodialysis (HD) was from Schiavon et al. (7). We observed lower PON1 in patients on standard HD and home nocturnal HD that was inversely correlated with C-reactive protein (CRP), a marker of inflammation (8). PON1 can be measured as both its activity and mass. In a cross-sectional study comparing a control group, patients on HD and patients with KT, Sztanek et al. (9) found PON1 activity was lowest for HD patients, followed by those with KT and highest in control subjects.

Most studies of patients on dialysis have measured PON1 enzymatic activity. Although reports are inconsistent, PON1 activity may be lowest for patients on dialysis, higher for patients that have received a KT, and highest for normal control subjects. This gradient in PON1 enzymatic activity is consistent with the gradient of kidney function. However, all studies have been cross-sectional in nature, with the exception of a study comparing change in arylesterase at 1, 6 and 12 months post-transplant (10). There has been no prospective determination of change in PON1 mass and activity with prospective restoration of kidney function through KT. There has also been no study that compared the change in PON1 mass or activity with change in LV indices.

## Methods

Recruitment of participants has been previously described (2, 11). Briefly, adult patients (18-75 years old) on HD or peritoneal dialysis (PD) being considered for KT were recruited at the time of their pre-KT assessment. Patients were assigned to one of 2 groups depending on their availability of a potential living kidney donor. The KT group consisted of HD or PD patients expected to receive a living donor KT within 2 months; the dialysis group consisted of patients without identified living donors and therefore not expected to receive a transplant for the next 24 months. The study sample size target of 42 subjects per group was based on detection of a 5 μg/ml change in adiponectin, with an expected attrition rate of 20%. Study assessments were performed at baseline, and either 12 months later while still on dialysis or 12 months post-KT. The predominant immunosuppressant regimen at 12 months post-KT was the combination of tacrolimus, mycophenolate and prednisone (*n* = 33), followed by tacrolimus and prednisone (*n* = 3), mycophenolate, prednisone and sirolimus (*n* = 2) and tacrolimus with mycophenolate (*n* = 1). Either basiliximab or thymoglobulin are used for induction, followed by tacrolimus 0.1 mg/kg/d adjusted to maintain a target trough level of 5–10 ng/ml. Mycophenolate is dosed at 720 mg per os twice daily, and prednisone is dosed initially at 1 mg/kg/d, tapered to 5 mg daily by 2 months post-transplant.

The methods for cardiac MRI (CMR) measurement of LV volume and LV mass have been described in detail (11). Briefly, CMR was performed with a 1.5-tesla whole-body magnetic resonance scanner (Intera: Philips Medical Systems, Best, The Netherlands) using a phased-array cardiac coil and retrospective vectorographic gating. All CMR studies were analyzed by readers blinded to the information about the patient and the time point of data acquisition. CMR data was analyzed by an experienced reader using cvi42 software (Circle Cardiovascular, Calgary, Canada). LV volumes and mass were allometrically adjusted by dividing by height in meters^2.7^ (12). Adiponectin was measured using the Meso Scale Discovery human adiponectin assay (#K151BXC-2, Meso Scale Diagnostics, Rockville, Maryland) and calibrated to the Millipore enzyme-linked immunoassay (#EZHADP-61-K, Millipore, St. Charles, MO, USA). N-terminal proB-type natriuretic peptide (NT-proBNP) was measured on the Roche Cobas 6000 601e module (Mississauga, ON, Canada). Measurement of PON1 was an a priori secondary variable. PON1 enzyme mass was measured using serum as described (13) with the modification that 4–20% Criterion sodium dodecylsulfate polyacrylamide gels (BioRad, Mississauga, ON, Canada) were used to separate serum proteins. A serum pool was analyzed in 11 separate runs, assigned a value of 108.8 μg/ml for PON1 concentration and used as a calibrator for all gels. PON1 Q192R phenotype was determined using high and low salt conditions with phenylacetate as a substrate and with 4-(chloromethyl)phenyl acetate as described by Richter et al. (14). We also measured blood hemoglobin and serum albumin concentrations, since they are important prognostic markers for survival in ESKD that improve with KT, as well as NT-proBNP due to its known decline post-KT and correlation with LV volumes. C-reactive protein (CRP) was measured as a marker of inflammation. Statistical analysis was done using SAS version 9.4 (Cary, NC, USA). Graphs were plotted using GraphPad Prism version 9.2. Between-group comparisons were done using unpaired *t*-test.

Since we recruited only patients with kidney failure who were kidney transplant candidates, we excluded those who were too ill to be considered for kidney transplantation. Therefore, the dialysis patients in this study represent the healthiest subgroup of dialysis patients. The dialysis patients' good baseline health may have suppressed any changes in their cardiac parameters over time, as might be seen with improved dialysis efficiency over time. We attempted to address this bias by excluding patients who were not yet on dialysis and receiving a pre-emptive kidney transplant, since those patients might be even healthier.

The study was approved by the Research Ethics Board at St. Michael's Hospital (REB 10-239) and by the ethics boards at the collaborating sites. All subjects provided written informed consent. The work described has been carried out in accordance with The Code of Ethics of the World Medical Association (Declaration of Helsinki) for experiments involving humans.

## Results

The baseline and 12-month characteristics of the dialysis group and transplant groups are shown in Table 1. Hemoglobin and albumin concentrations were significantly different between HD and PD patients at baseline. Therefore, a *post-hoc* sensitivity analysis was performed and the univariate analyses are presented separately by baseline dialysis type. Within dialysis type, baseline values for the dialysis vs. transplant subjects were not significantly different, with the exception of LV volume at diastole (ml/m^2.7^) for the PD patients. Allometrically adjusted LV mass (g/m^2.7^) was not different between the two groups. KT resulted in significant increases in hemoglobin concentration for both HD and PD patients, but KT significantly increased albumin concentration only in the PD patients. CRP concentration did not differ between HD and PD patients and was not significantly affected by KT.

**Table 1:** Characteristics of study subjects by treatment group and baseline dialysis modality.

	**Hemodialysis at baseline**		***p*-value**	**Peritoneal dialysis at baseline**		***p*-value**
	**Dialysis**	**Transplant**		**Dialysis**	**Transplant**	
Women/Men	8/23	10/17		4/8	2/10	
Age, yrs	54.1 ± 9.6	46.3 ± 12.1	0.008	59.2 ± 13.8	47.1 ± 13.7	0.04
**Baseline**						
Serum Creatinine μmol/L	674 ± 229	694 ± 230	0.75	789 ± 199	926 ± 332	0.23
BMI, kg/m^2^	27.2 ± 5.2	25.5 ± 4.9	0.2	25.7 ± 4.1	27.1 ± 3.6	0.39
Hemoglobin, g/L	118.7 ± 15.3^∧^	121.5 ± 13.4^*^	0.46	109.3 ± 11.7	106.7 ± 17.0	0.67
Total Chol, mM	4.0 ± 1.3	4.16 ± 1.22	0.63	4.15 ± 1.04	4.21 ± 1.11	0.89
Triglycerides, mM	1.46 ± 0.85	1.93 ± 1.39	0.12	1.53 ± 0.53	2.03 ± 0.84	0.10
LDL Chol, mM	2.17 ± 1.12	2.17 ± 0.97	0.98	2.35 ± 1.0	2.38 ± 0.95	0.93
HDL Chol, mM	1.19 ± 0.33	1.13 ± 0.32	0.51	1.11 ± 0.36	0.90 ± 0.19	0.10
Albumin, g/L	41.6 ± 4.2^#^	41.7 ± 3.8^§^	0.91	35.8 ± 2.4	36.9 ± 3.5	0.40
CRP, mg/L	6.5 ± 10.2	3.8 ± 5.0	0.21	6.5 ± 7.3	6.0 ± 6.0	0.88
LV systolic volume, ml/m^2.7^	15.9 ± 5.5	17.5 ± 6.5	0.33	15.2 ± 5.0	19.3 ± 5.8	0.08
LV diastolic volume, ml/m^2.7^	39.2 ± 10.2	40.6 ± 10.7	0.63	37.0 ± 7.8	45.0 ± 9.4	0.036
LV mass index, g/m^2.7^	30.8 ± 9.9	29.2 ± 8.6	0.50	28.7 ± 4.5	31.4 ± 8.2	0.32
Ln NT-proBNP, pg/ml	7.49 ± 1.55	7.23 ± 1.27	0.50	6.81 ± 1.38	7.30 ± 1.37	0.41
Adiponectin, μg/ml	23.0 ± 13.5	20.6 ± 11.0	0.47	20.4 ± 10.7	27.5 ± 10.8	0.13
PON1 μg/ml	98.2 ± 24.2	92.0 ± 22.9	0.32	80.8 ± 19	79.5 ± 26	0.89
PON1 arylesterase, U/ml	88.1 ± 19.2	82.3 ± 19.5	0.25	82.2 ± 19.5	78.4 ± 19.7	0.65
PON1 specific activity, U/μg	0.91 ± 0.16	0.91 ± 0.18	0.99	1.02 ± 0.11	1.02 ± 0.18	0.92
**Month 12**						
Serum creatinine μmol/L	757 ± 234	129 ± 103	<0.0001	696 ± 177	118 ± 31	<0.0001
BMI, kg/m^2^	27.6 ± 5.7	26.7 ± 4.7	0.49	25.0 ± 3.4	28.2 ± 5.4	0.11
Hemoglobin, g/L	116.6 ± 12.1	134.3 ± 20.3	0.0003	113.1 ± 13.3	137.6 ± 18.6	0.0012
Total Chol, mM	3.98 ± 1.32	4.39 ± 1.13	0.21	3.94 ± 1.07	4.33 ± 1.17	0.40
Triglycerides, mM	1.56 ± 0.96	1.85 ± 1.38	0.34	1.54 ± 0.58	1.90 ± 0.87	0.24
LDL Chol, mM	2.08 ± 0.93	2.33 ± 0.81	0.29	2.18 ± 0.90	2.14 ± 0.72	0.90
HDL Chol, mM	1.13 ± 0.37	1.24 ± 0.39	0.29	1.05 ± 0.29	1.19 ± 0.28	0.25
Albumin, g/L	42.8 ± 3.5	43.6 ± 2.9	0.36	36.5 ± 2.75	43.5 ± 3.0	<0.0001
CRP, mg/L	4.1 ± 3.9	3.0 ± 4.0	0.32	9.7 ± 13.3	4.8 ± 4.9	0.26
LV systolic volume, ml/m^2.7^	15.5 ± 5.2	14.5 ± 4.4	0.47	15.4 ± 11.4	14.7 ± 12.8	0.72
LV diastolic volume, ml/m^2.7^	39.6 ± 10.4	36.9 ± 8.1	0.29	37.3 ± 29.5	37.1 ± 34.6	0.96
LV mass index, g/m^2.7^	30.2 ± 9.5	28.0 ± 6.0	0.29	28.5 ± 8.9	27.4 ± 4.5	0.70
Ln NT-proBNP, pg/ml	7.74 ± 1.53	4.81 ± 1.28	<0.0001	7.40 ± 1.77	4.95 ± 1.25	0.0007
Adiponectin μg/ml	21.7 ± 13.4	15.1 ± 7.1	0.021	23.3 ± 17.2	18.1 ± 11.3	0.39
PON1 μg/ml	96.2 ± 21.1	106.8 ± 22.4	0.07	83.0 ± 17.1	100.3 ± 23.8	0.06
PON1 arylesterase, U/ml	88.1 ± 19.9	99.2 ± 19.5	0.038	82.5 ± 17.0	95.4 ± 26.4	0.18
PON1 specific activity, U/μg	0.93 ± 0.2	0.95 ± 0.16	0.80	1.00 ± 0.13	0.95 ± 0.19	0.46

*Baseline hemoglobin:*

* *p = 0.006 for hemodialysis vs. peritoneal dialysis for the transplant group;*

∧ *p = 0.06 for hemodialysis vs. peritoneal dialysis for the dialysis group; Baseline albumin:*

# *p = 0.0011 for hemodialysis vs. peritoneal dialysis for the transplant group;*

§ *p < 0.0001 for hemodialysis vs. peritoneal dialysis for the dialysis group*.

KT increased PON1 mass for both HD and PD patients, with near-significant *p* values of 0.07 and 0.06, respectively (Table 1). Univariate analyses of PON1 mass and activity comparing the dialysis and KT groups is shown in Table 2. The data for the HD and PD subjects within group was combined for this analysis. ESKD baseline values were not significantly different between the dialysis and KT groups, but at 12 months the absolute values for PON1 activity (*p* = 0.0125) and PON1 mass (*p* = 0.0119) were significantly higher in the KT group whether analyzed as the change in activity or mass (*p* < 0.0001 for each). Notably, the standard deviation for the dialysis group and the transplant group was similar.

**Table 2:** Paraoxonase 1 mass and activity by treatment group.

	**Dialysis**	**Transplant**	**p-value**
n	43	38	
**Baseline**		
PON1 arylesterase activity, U/ml	86.4 ± 19.3	81.2 ± 19.4	0.22
PON1 mass, μg/ml	93.4 ± 24.0	88.6 ± 24.1	0.35
PON1 specific activity, U/μg	0.94 ± 0.16	0.94 ± 0.18	0.99
**Month 12**			
PON1 arylesterase activity, U/ml	86.6 ± 19.1	98.09 ± 21.4	0.0125
PON1 mass, μg/ml	92.5 ± 20.7	104.9 ± 22.7	0.0119
PON1 specific activity, U/μg	0.95 ± 0.18	0.95 ± 0.16	0.89
Change PON1 arylesterase, U/ml	0.15 ± 10.5	16.9 ± 13.8	<0.0001
Change PON1 mass, μg/ml	−0.84 ± 13.3	16.6 ± 15.8	<0.0001

Table 3 provides an analysis of differences between HD and PD patients by analysis of change in cardiovascular biomarkers. The most striking impact of dialysis modality was on change in albumin, which was highly significant for PD patients (*p* = 0.0005), but not HD patients (*p* = 0.34). The change in systolic and diastolic LV volume was greater for subjects on PD and reached statistical significance despite the smaller sample size. The decrease in systolic and diastolic LV volume seen for HD patients was consistent with a beneficial, albeit smaller, effect of KT for this group. The change in PON1 activity and mass was significant for both HD and PD patients. The change in hemoglobin was also significant for both dialysis modalities, although it was greater for the PD patients. The change in HDL cholesterol was also found to be significant for both HD and PD patients. Thus, among the measured liver products, albumin was distinguished from PON1 and HDL cholesterol as being strongly affected by baseline dialysis modality.

**Table 3:** Change in paraoxonase-1 mass and activity and relationship with hemoglobin and albumin by baseline dialysis modality.

	**Hemodialysis at baseline**		***p*-value**	**Peritoneal dialysis at baseline**		***p*-value**
	**Dialysis**	**Transplant**		**Dialysis**	**Transplant**	
n	*N* = 28	*N* = 26		*N* = 12	*N* = 11	
Change in albumin, g/L	1.04 ± 3.81	2.11 ± 4.41	0.34	0.67 ± 2.74	6.9 ± 4.4	0.0005
n	*N* = 30	*N* = 27		*N* = 12	*N* = 11	
Change in LV systolic volume, ml/m^2.7^	−0.43 ± 4.09	−2.93 ± 5.91	0.067	0.28 ± 5.1	−4.42 ± 4.76	0.033
Change in LV diastolic volume, ml/m^2.7^	0.28 ± 9.3	−3.63 ± 7.98	0.096	0.33 ± 9.2	−7.91 ± 9.45	0.046
n	*N* = 31	*N* = 27		*N* = 12	*N* = 11	
Change in PON1 activity, U/ml	0.07 ± 11.0	16.9 ± 13	<0.0001	0.35 ± 9.46	16.9 ± 16.2	0.0065
n	*N* = 31	*N* = 27		*N* = 12	*N* = 11	
Change in PON1 mass, μg/ml	−2.01 ± 12.9	14.9 ± 16.9	<0.0001	2.2 ± 14.6	20.8 ± 12.32	0.0036
Change PON1 specific activity, U/μg	0.019 ± 0.12	0.03 ± 0.09	0.69	−0.018 ± 0.1	−0.061 ± 0.14	0.4
n	*N* = 31	*N* = 27		*N* = 12	*N* = 12	
Change in Hemoglobin, g/L	−2.1 ± 16.8	12.8 ± 23.2	0.0066	3.8 ± 12.1	30.9 ± 29.7	0.0106
Change HDL cholesterol, mM	−0.05 ± 0.22	0.16 ± 0.29	0.0081	−0.05 ± 0.18	0.29 ± 0.3	0.0026

**Figure 1:**
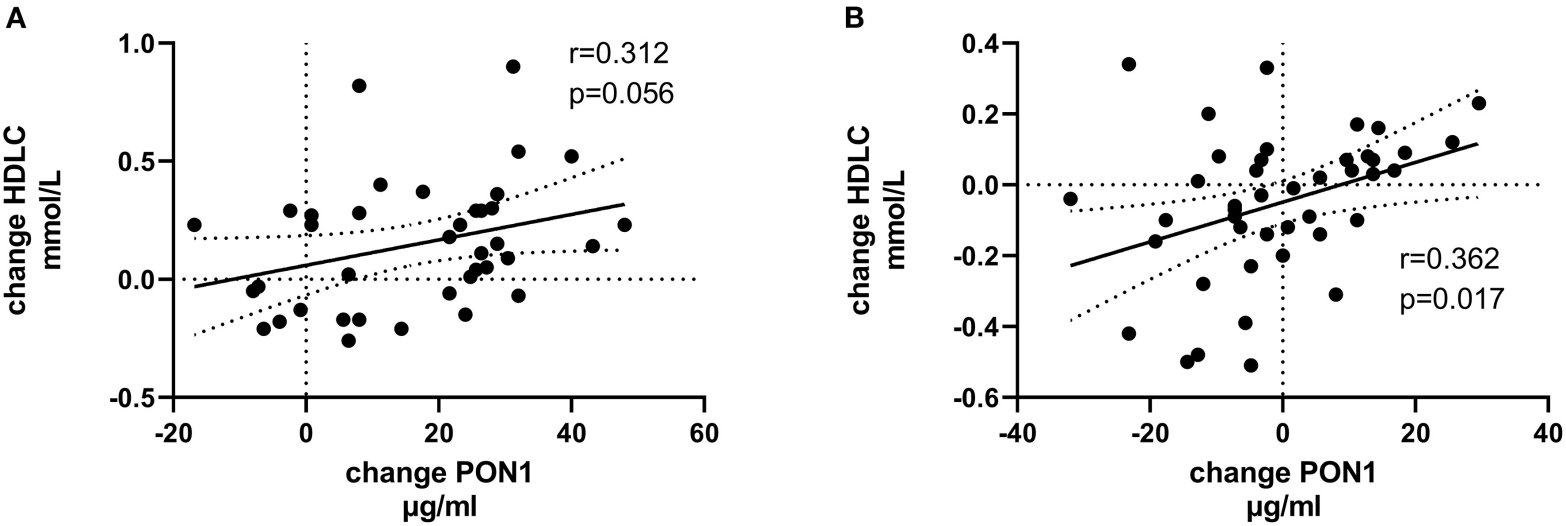
Change in HDLC vs. change in PON1 mass. **(A)** Transplant group. **(B)** Dialysis group.

**Figure 2:**
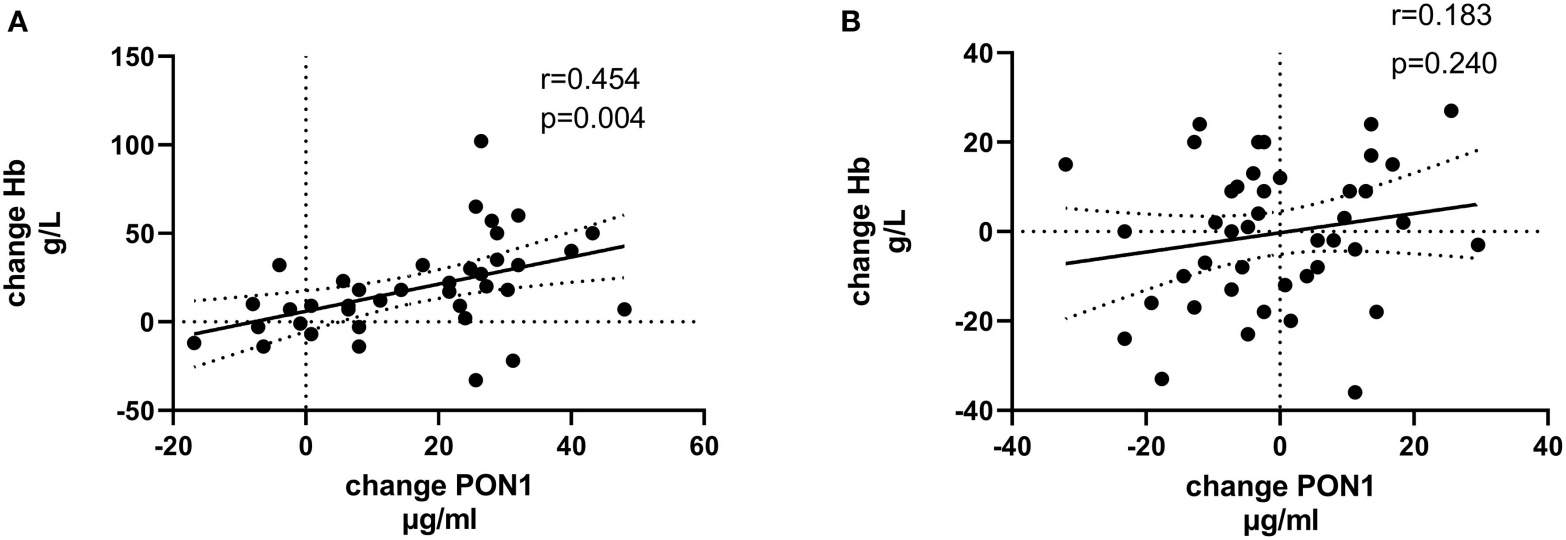
Change in Hb vs. change in PON1 mass. **(A)** Transplant group. **(B)** Dialysis group.

**Figure 3:**
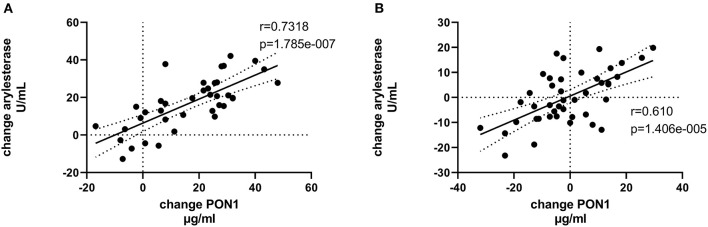
Change in PON1 arylesterase activity vs. change in PON1 mass. **(A)** Transplant group. **(B)** Dialysis group.

**Figure 4:**
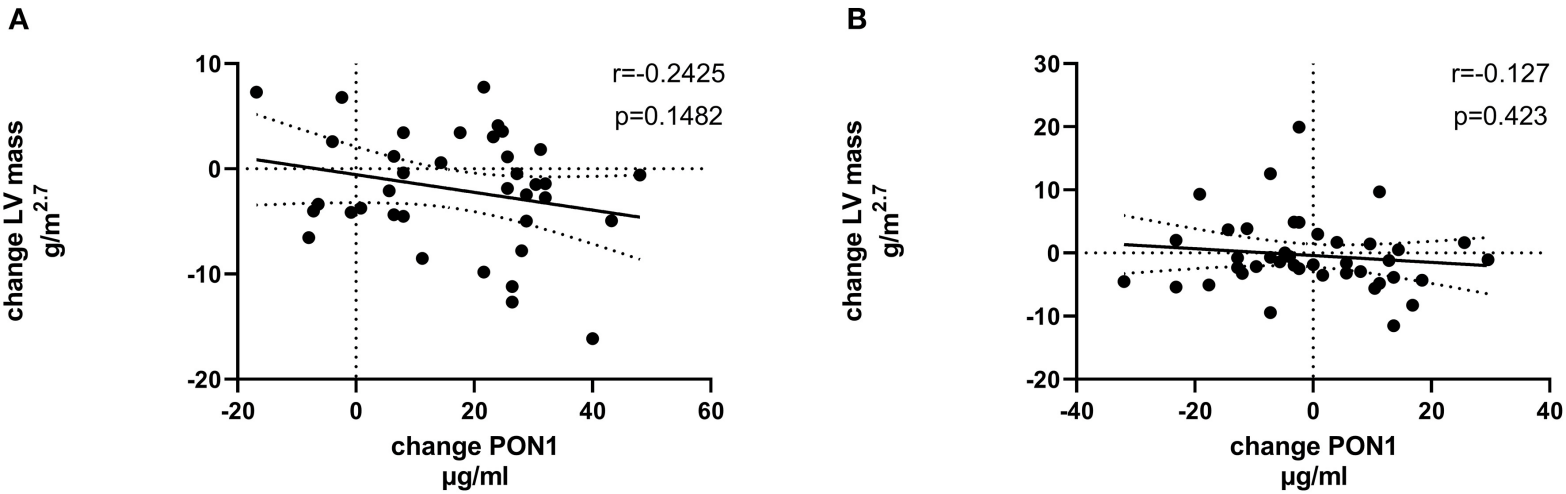
Change in LV mass vs. change in PON1 mass. **(A)** Transplant group. **(B)** Dialysis group.

**Figure 5:**
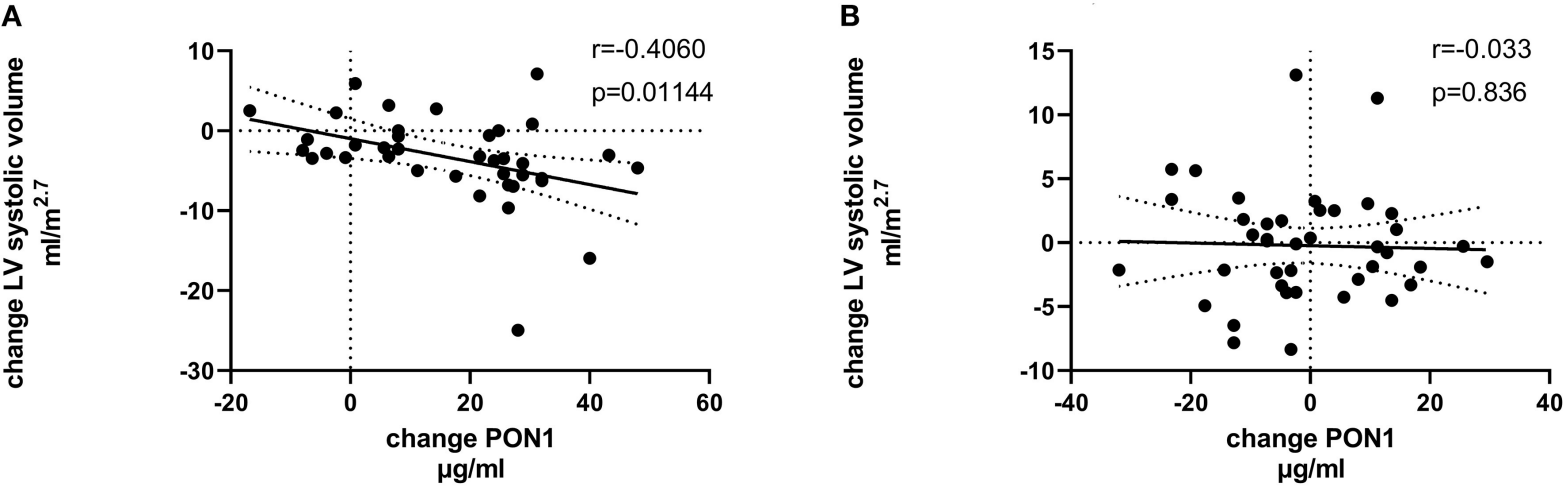
Change in LV systolic volume vs. change in PON1 mass. **(A)** Transplant group. **(B)** Dialysis group.

**Figure 6:**
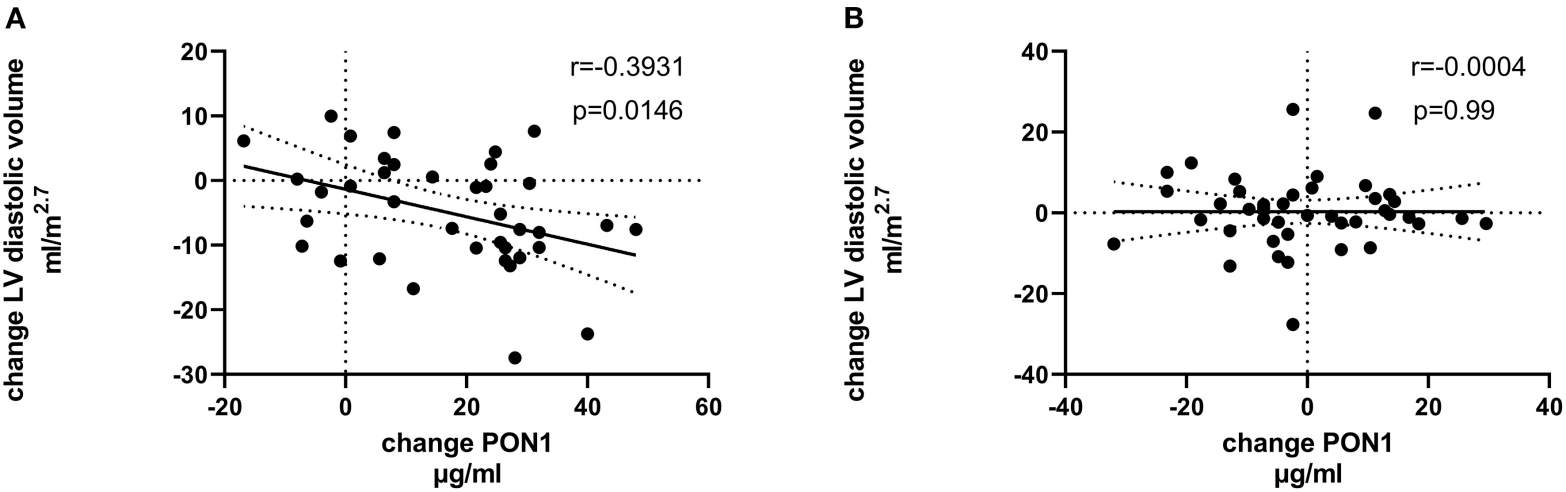
Change in LV diastolic volume vs. change in PON1 mass. **(A)** Transplant group. **(B)** Dialysis group.

**Figure 7:**
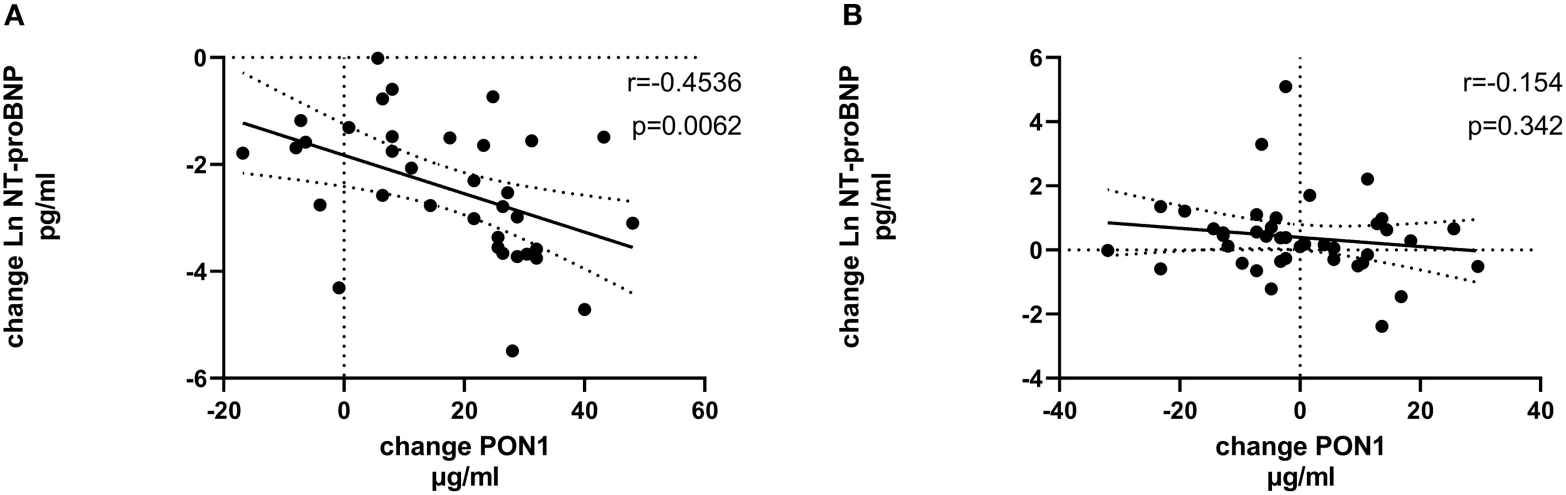
Change in Ln NT-proBNP vs. change in PON1 mass. **(A)** Transplant group. **(B)** Dialysis group. Change was calculated as the month 12 value minus the baseline value for each variable. Values shown in the figures are the Pearson correlation coefficient and the *p*-value. LV, left ventricular; Hb, hemoglobin. LV, left ventricular; LnNT-proBNP, natural log of NT-proB-type natriuretic peptide; PON1, paraoxonase 1; HDLC, high density lipoprotein cholesterol.

Tables 4, 5 (and shown in Figures 1–7) provide correlation analyses for PON1 mass and activity, HDL cholesterol, adiponectin, NT-proBNP, LV systolic and diastolic volume, and LV mass for the KT group and the dialysis group, respectively. The data for the HD and PD subjects was combined for this analysis. For the KT group, the change in PON1 mass and arylesterase activity were significantly correlated (*p* < 0.0001). The change in hemoglobin was also positively correlated with change on PON1 mass (*p* = 0.0042), but not with PON1 activity (*p* = 0.275). Further, the change in PON1 mass was inversely correlated with the change in LV diastolic volume (*p* = 0.0146), systolic volume (*p* = 0.0114) and Ln NT-proBNP (*p* = 0.0062). Although the change in PON1 mass was correlated with the change in HDL cholesterol concentration (*p* = 0.0562), the change in HDL cholesterol was not significantly correlated with LV volume or mass. The change in HDL cholesterol was significantly correlated with the change in PON1 arylesterase (*p* = 0.0003) and adiponectin (*p* = 0.0062). Thus, the change in PON1 mass has a unique association with LV changes, unlike HDL cholesterol and PON1 arylesterase activity.

**Table 4:** Pearson correlation of change in left ventricular measurements and hemoglobin with PON1, HDL cholesterol, and adiponectin for subjects receiving a kidney transplant.

**Transplant group**	**LV diastolic volume**	**LV systolic volume**	**LVMI^**2.7**^**	**Hemoglobin g/L**	**LnNT-proBNP pg/ml**	**PON1 μg/ml**	**PON1 units/ml**	**HDLC mM**	**Adiponectin μg/ml**
LV_diastolic	1	0.846	0.7188	−0.4969	0.6074	−0.3931	−0.1294	−0.0116	0.3019
Volume, ml/m^2.7^		<0.0001	<0.0001	0.0015	0.0001	0.0146	0.4387	0.9452	0.0654
	38	38	37	38	35	38	38	38	38
LV_systolic		1	0.5729	−0.5379	0.6251	−0.406	−0.3002	−0.0912	0.2039
Volume, ml/m^2.7^			0.0002	0.0005	<0.0001	0.0114	0.0671	0.5859	0.2195
		38	37	38	35	38	38	38	38
LVMI, g/m^2.7^			1	−0.4171	0.3962	−0.2425	−0.1157	−0.0485	0.2169
				0.0102	0.0204	0.1482	0.4962	0.7754	0.1972
			37	37	34	37	37	37	37
Hemoglobin				1	−0.385	0.4536	0.1816	0.0661	−0.2836
g/L					0.0224	0.0042	0.2751	0.6892	0.0845
				39	35	38	38	39	38
LnNT-proBNP, pg/ml					1	−0.4536	−0.3774	−0.0572	0.3865
						0.0062	0.0254	0.7442	0.0218
					35	35	35	35	35
PON1, μg/ml						1	0.7318	0.3125	−0.1513
							<0.0001	0.0562	0.3647
						38	38	38	38
PON1, units/ml							1	0.5572	0.2414
								0.0003	0.1443
							38	38	38
HDLC, mM								1	0.4363
									0.0062
								39	38
Adiponectin, μg/ml									1
									38

*Values shown in the table are the Pearson correlation coefficient, the p-value, and the number of subjects. LV, left ventricular; LVMI, left ventricular mass index; LnNT-proBNP, natural log of NT-proB-type natriuretic peptide; PON1, paraoxonase 1; HDLC, high density lipoprotein cholesterol*.

**Table 5:** Pearson correlation of change in left ventricular measurements and hemoglobin with PON1, HDL cholesterol, and adiponectin for subjects on dialysis.

**Dialysis group**	**LV diastolic volume**	**LV systolic volume**	**LVMI^**2.7**^**	**Hemoglobin g/L**	**LnNT-proBNP pg/ml**	**PON1 μg/ml**	**PON1 units/ml**	**HDLC mM**	**Adiponectin μg/ml**
LV_diastolic	1	0.8316	0.4975	−0.3676	0.5817	−0.0004	−0.3591	0.1654	0.2458
Volume, ml/m^2.7^		<0.0001	0.0008	0.0166	0.0001	0.9981	0.0195	0.2951	0.1166
	42	42	42	42	39	42	42	42	42
LV_systolic		1	0.6079	−0.3469	0.6212	−0.0329	−0.27	0.0734	0.3768
Volume, ml/m^2.7^			<0.0001	0.0244	<0.0001	0.8363	0.0837	0.6443	0.0139
		42	42	42	39	42	42	42	42
LVMI, g/m^2.7^			1	−0.3493	0.7602	−0.127	−0.2118	−0.0151	0.461
				0.0234	<0.0001	0.423	0.1782	0.9243	0.0021
			42	42	39	42	42	42	42
Hemoglobin,				1	−0.3371	0.183	0.2703	0.2573	0.1063
g/L					0.0334	0.2402	0.0796	0.0958	0.4975
				43	40	43	43	43	43
LnNT-proBNP, pg/ml					1	−0.1542	−0.3183	−0.1224	0.3963
						0.3421	0.0453	0.4519	0.0114
					40	40	40	40	40
PON1, μg/ml						1	0.61	0.3619	0.1381
							<0.0001	0.0171	0.3771
						43	43	43	43
PON1, units/ml							1	0.4	0.1757
								0.0082	0.2597
							43	43	43
HDLC, mM								1	0.3252
									0.0333
								43	43
Adiponectin, μg/ml									1
									43

*Values shown in the table are the Pearson correlation coefficient, the p-value and the number of subjects. LV, left ventricular; LVMI, left ventricular mass index; LnNT-proBNP, natural log of NT-proB-type natriuretic peptide; PON1, paraoxonase 1; HDLC, high density lipoprotein cholesterol*.

The median percent change in PON1 mass was −3.9 vs. 20.3% for the dialysis and KT groups, respectively. The change in PON1 mass was not correlated with the average PON1 mass (*p* = 0.56), indicating that baseline PON1 concentrations were not a determinant of the response to KT. Thus, the incremental increase in PON1 mass was similar across the PON1 concentration range. Only the change in Ln NT-proBNP was significantly different from the null (*p* = 0.0487) in the dialysis group. The correlation of PON1 mass and arylesterase activity was significant across groups (*p* < 0.0001). Consistent with these two measures having different determinants in the dialysis group (Table 5), PON1 arylesterase variation correlated inversely with the change in LV diastolic volume (*p* = 0.019) and with the change in Ln NT-proBNP (*p* = 0.045), whereas there was no correlation of the change in PON1 mass with these in the dialysis group (*p* = 0.998, 0.342, respectively).

PON1 Q192R phenotype frequency was not different between the dialysis and KT groups (Supplementary Table 1, Fishers exact test *p* = 0.74). Further, PON1 Q192R phenotype was not a significant factor for the change in PON1 mass (data not shown).

## Discussion

We have shown for the first time that the increase in PON1 mass post-KT inversely correlates with change in LV volume and positively correlates with the change in hemoglobin. The current study also prospectively, rather than cross-sectionally, confirms that PON1 activity is higher with greater kidney function. Further, we have documented that change in PON1 mass post-KT is independent of baseline concentration or inflammation. Separating PON1 mass change from change in CRP, HDL cholesterol and albumin supports specific effects that link PON1, hemoglobin and LV volume. PON1 activity, influenced by HDL cholesterol, does not uniquely share a common factor with change in hemoglobin or LV volume.

The cause-effect relationship among change in LV volume, change in hemoglobin and change in PON1 mass cannot be elucidated from the current study design. Several candidates are suggested by the current literature to explain these relationships. PON1 has been shown to be involved, as a lactonase, in the metabolic conversion of 5,6-epoxyeicosatrienoic acid (15). The epoxyeicosatrienoic acids are among the molecules that regulate response to mechano-transduction as regulators of TRPV4 (16, 17). Independently, the production of B-type natriuretic peptide, as reflected by the inverse relationship of PON1 with NT-proBNP, could indicate a link between PON1 and mechano-sensing pathways. Thus, one could speculate that PON1 mass reflects a role in response to left ventricular volume. The positive correlation of the change in PON1 mass with the change in hemoglobin could indicate that factors involved in iron metabolism regulate PON1 gene expression in concert with regulating hemoglobin concentration. This would be consistent with an increase in PON1 in response to erythropoietin therapy that has been reported in a single study (18). However, currently there is no information that would link the regulation of human PON1 gene expression and iron metabolism.

Despite a greater understanding of PON1 mass, the importance of reduced PON1 activity seen in states of reduced kidney function and partially restored kidney function remains to be established. Bhattacharyya et al. (19) reported that PON1 Q192R genotype, paraoxonase and arylesterase activities were related to cardiovascular outcomes in a cohort of subjects undergoing elective diagnostic cardiac catheterization. The lowest quartile of arylesterase activity had a 4.5-fold hazard ratio for nonfatal myocardial infarction or cerebrovascular accident. Hammadah et al. (20) reported event-free survival over 5 years for subjects with chronic heart failure from the Atlanta Cardiomyopathy Consortium. BNP was highest, and HDL cholesterol was lowest, for the lowest tertile of arylesterase activity. The lowest tertile of baseline arylesterase activity was associated with a 2.6 hazard ratio for adverse events.

PON1 has a well-recognized labile metal binding site, in addition to its enzymatic activity. This labile site requires calcium for enzyme activity, but it can also bind metals. Thus, one potential function of PON1 is in the regulation of iron, zinc and other divalent cations (21–23). Rahimi-Ardabili et al. (24) reported that zinc sulfate supplementation increased HDL cholesterol, apoAI and paraoxonase activity in patients on HD, but that this response was not specific to paraoxonase and may reflect a general effect on HDL production or clearance. This putative function of PON1 would be dependent upon its mass and would have the potential to not only be independent of the lactonase activity but inverse to such activity.

KT recipients benefit markedly from their improved kidney function compared to remaining on dialysis, experiencing improved cardiovascular outcomes (25) although outcome depends on the amount of kidney function restored (26). While reduced LV volume and increased hemoglobin are well-known effects of KT, the relationship of these improvements to improved cardiovascular outcomes remains unestablished. In this study, we have shown that PON1 mass especially, and possibly PON1 activity, may be key mediators in the pathway linking restored kidney function and improved cardiovascular outcomes. The current study generates the hypothesis that interventions selectively directed toward increasing PON1 mass and activity may help provide further insight into the mechanisms that connect the kidney and heart in patients with pathological cardiorenal syndromes, such as those with ESKD.

The study is limited by its inclusion of largely healthy patients, since those with recent (<6 months) cardiac events were also excluded. Most transplant patients received living donor transplants, which have the best outcomes with less acute tubular necrosis, acute rejection, and cardiac events. This may amplify any differences from the dialysis group. The magnitude is expected to be small since additionally, 12 months of follow-up is expected to be sufficient to overcome these challenges to the transplant, before the follow-up CMR. Fortunately, no patients in this study, transplant or dialysis, experienced cardiac events. The findings from this study cannot be generalized to deceased donor transplants, which constitute about 60% of all transplants and are subject to more complications.

## Data Availability Statement

The raw data supporting the conclusions of this article will be made available by the authors, without undue reservation.

## Ethics Statement

The studies involving human participants were reviewed and approved by St. Michael's Hospital Research Ethics Board, University Health Network Research Ethics Board, and the London Health Sciences Centre Research Ethics Board. The patients/participants provided their written informed consent to participate in this study.

## Author Contributions

GP, PC, and AY: study conception and design and data acquisition. GP, PC, MN, RW, LG, CL, AK, and AY: data analysis and interpretation and drafting and revision of the manuscript. Each author contributed important intellectual content during manuscript drafting or revision, accepts personal accountability for the author's own contributions, and agrees to ensure that questions pertaining to the accuracy or integrity of any portion of the work are appropriately investigated and resolved. All authors contributed to the article and approved the submitted version.

## Funding

This study was funded by the Heart and Stroke Foundation of Canada, Grant Number HSFNA7077 and the Canadian Institutes for Health Research, Grant Number 136954.

## Conflict of Interest

The authors declare that the research was conducted in the absence of any commercial or financial relationships that could be construed as a potential conflict of interest.

## Publisher's Note

All claims expressed in this article are solely those of the authors and do not necessarily represent those of their affiliated organizations, or those of the publisher, the editors and the reviewers. Any product that may be evaluated in this article, or claim that may be made by its manufacturer, is not guaranteed or endorsed by the publisher.
