# Supplementary material for: The Increase in Paraoxonase 1 Is Associated With Decrease in Left Ventricular Volume in Kidney Transplant Recipients
Source: Front Cardiovasc Med. 2021 Dec 2;8:763389. doi: 10.3389/fcvm.2021.763389 (PMC8674585; doi:10.3389/fcvm.2021.763389)
Supplement: Supplementary file 1 [file Table_1.docx]

Supplementary Table 1.

Table S1. Paraoxonase 1 phenotype distribution. Fisher’s exact test p=0.743.

| PON1 192 phenotype | Gln/Gln (N) | Gln/Arg (N) | Arg/Arg (N) |
| --- | --- | --- | --- |
| Dialysis | 12 | 21 | 10 |
| Transplant | 14 | 16 | 8 |
